# Pleural-Based Intrathoracic Cystic Lymphangioma in an Infant Mimicking a Pneumonia

**DOI:** 10.1155/2019/7920591

**Published:** 2019-05-06

**Authors:** Zev Frimer, Daniel Fink, Ruth Cytter-Kuint, Victoria Doviner, Elie Picard

**Affiliations:** ^1^Pediatric Pulmonary Unit, Shaare Zedek Medical Center, Affiliated to the Hebrew University, School of Medicine, Jerusalem, Israel; ^2^Cardiothoracic Surgery Department, Shaare Zedek Medical Center, Affiliated to the Hebrew University, School of Medicine, Jerusalem, Israel; ^3^Radiology Department, Shaare Zedek Medical Center, Affiliated to the Hebrew University, School of Medicine, Jerusalem, Israel; ^4^Pathology Department, Shaare Zedek Medical Center, Affiliated to the Hebrew University, School of Medicine, Jerusalem, Israel

## Abstract

Cystic lymphangioma is an uncommon benign tumor that occurs primarily in children in the cervical region. We report the first case of a pleural-based cystic lymphangioma in an infant. The patient was admitted for RUL pneumonia. Because of the persistence of the radiographic findings despite clinical improvement, a computed tomography (CT) and a magnetic resonance imaging (MRI) scan were performed. They showed a multiloculated cystic lesion in the superior posterior right hemithorax. A surgical procedure was performed with complete resection of the tumor. Histopathological examination showed a pleural-based intrathoracic multicystic lymphangioma. One year after the surgery, the patient feels well without any sign of recurrence.

## 1. Introduction

Cystic lymphangioma is a rare congenital malformation first described in 1843 [1] which results from failure of a primary lymphatic sac to establish drainage into the venous system. Almost 90% of cases are diagnosed within the first 2 years of life [2]. These cysts can affect any site in the body although less than 1% of lymphangiomas are intrathoracic [3]. Very few of them have a pleural basis and are named “pleural-based lymphangioma.” To the best of our knowledge, they have been reported only twice, one in an adult [4] and one in a teenager [2]. We hereby present the first case of an infant with a pleural-based intrathoracic lymphangioma.

## 2. Case Report

**Figure 1:**
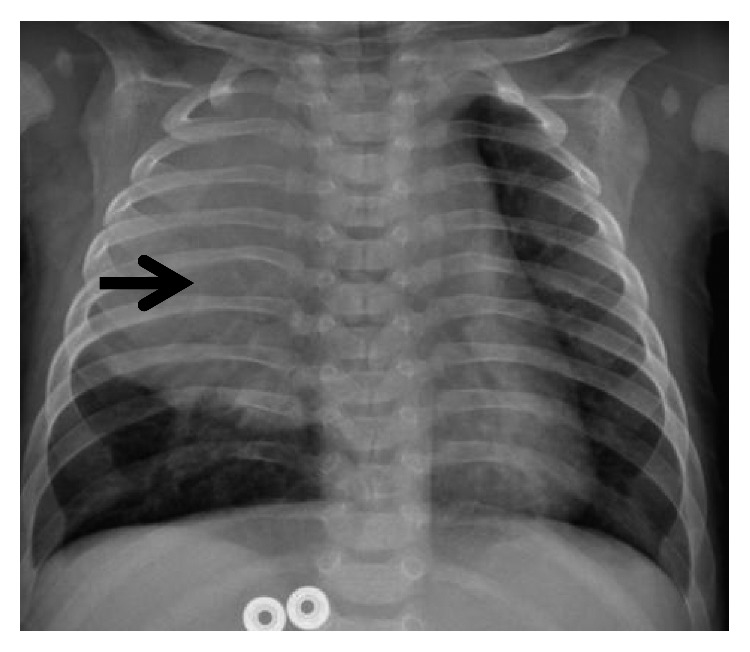
Chest X-ray: RUL infiltration and enlarged intercostal space in level T6-T7 on the same side.

**Figure 2:**
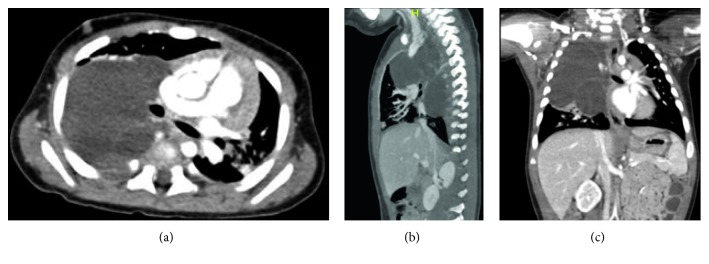
Contrast enhanced computed tomography (CT): a large multicystic lesion in the right hemithorax with mediastinal shifting. The bronchus and azygous are not affected.

**Figure 3:**
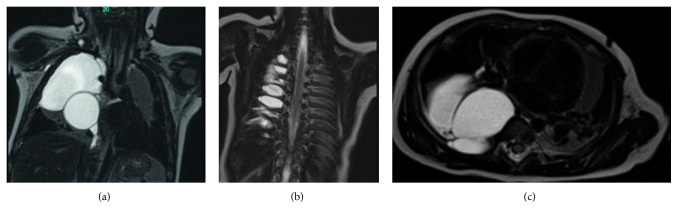
Magnetic resonance imaging (MRI): insinuating multiloculated cystic lesion. (a) Small extrapleural extensions of the cystic mass superiorly and laterally. (b) The mass bulges in the posterior intercostal spaces, without neuroforaminal involvement. (c) Posterior intercostal bulging, with no neuroforaminal involvement, and hilar structures are not compressed.

**Figure 4:**
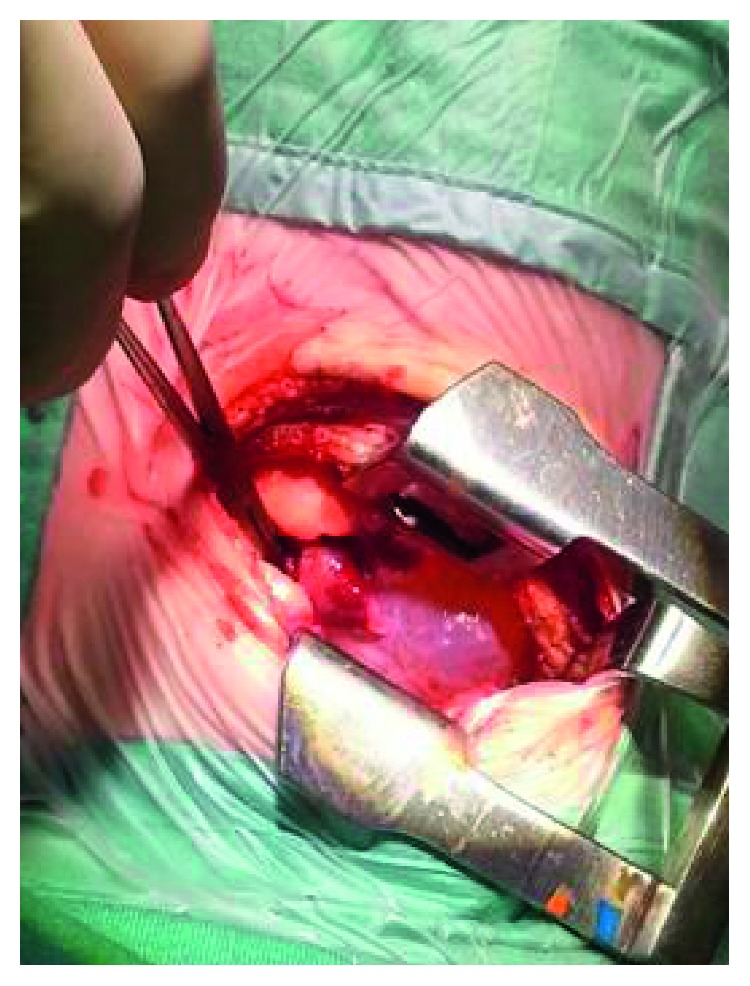
Perioperative picture showing intrathoracic cystic lymphangioma.

**Figure 5:**
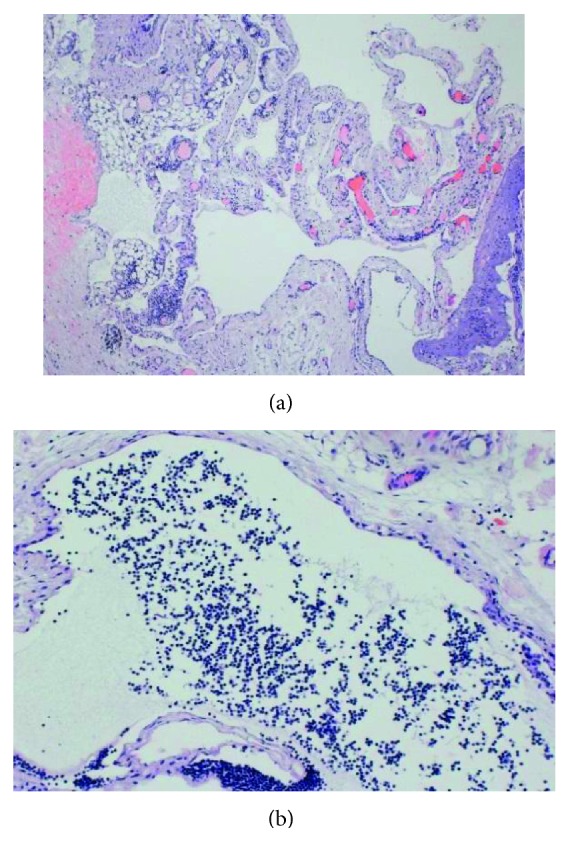
Microscopic features of the cyst (H&E staining). (a) Panoramic view of the multicystic tumor (original magnification ×12.5). (b) Proteinatious fluid and small lymphocytes in the lumen (original magnification ×40).

**Figure 6:**
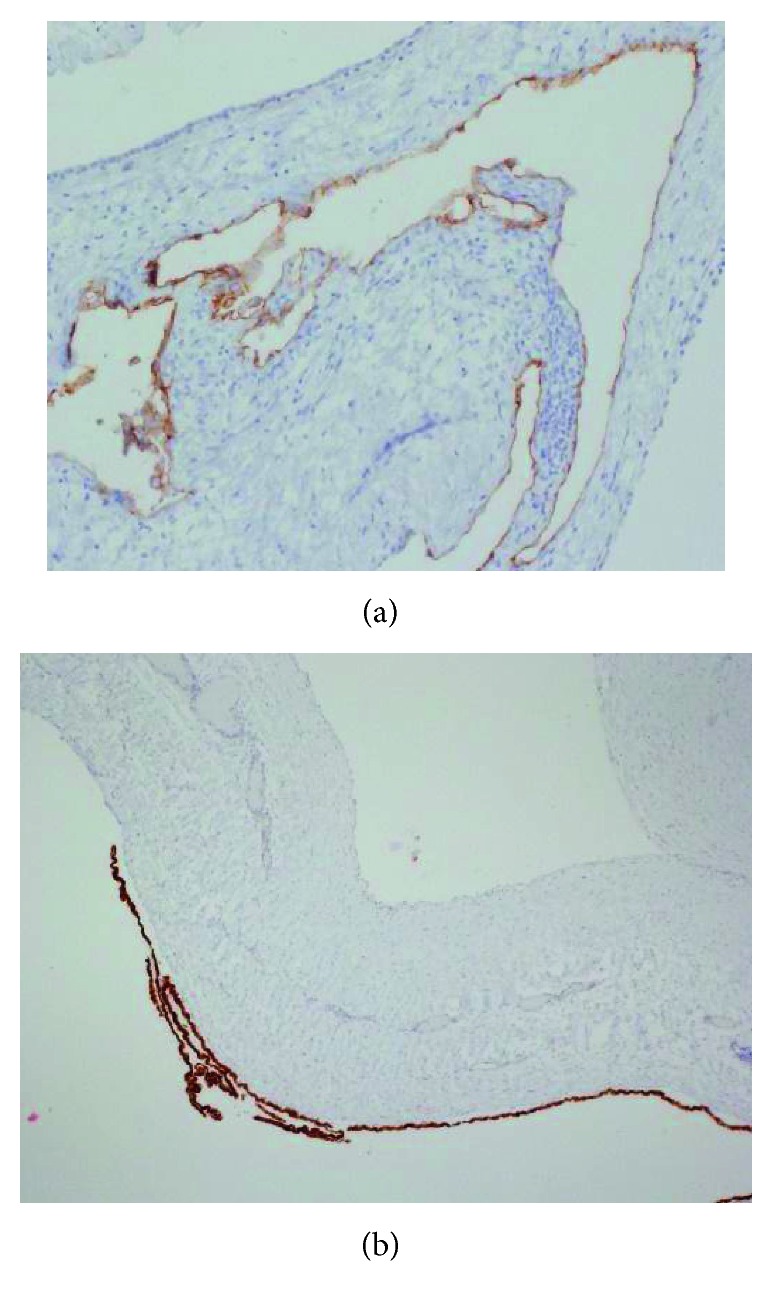
Immunohistochemical findings. (a) The cells lining the inner surface of the cystic lesion are positive for D2-40 staining, expressed in lymphatic endothelium (original magnification ×12.5). (b) The cells lining the outer surface of the cystic lesion are positive for calretinin staining, expressed in mesothelial cells (original magnification ×12.5).

A 4-month-old boy, with a healthy perinatal background, was referred to our emergency room because of five days of fever, cough, and dyspnea. Until this event, his medical history was unremarkable. Physical examination on admission showed dullness on percussion and decreased intensity of breath sounds in the right hemithorax. Blood count revealed leukocytosis (29,400/*μ*l with 61% PMN) and elevated C-reactive protein level (5.43 mg/dl (normal < 0.5)). Chest X-ray demonstrated a large infiltration in the right upper lobe (Figure 1). Hence, with a diagnosis of right lobar pneumonia, the child was hospitalized and managed with antibiotics. Under antibiotic treatment, the patient had clinical and laboratory improvement. A follow-up X-ray done a few days later showed similar findings without significant change. Moreover, due to the increased intercostal space between T6-T7 on the right (Figure 1), the possibility of slow growing extrapleural mass was suggested. Computed tomography (CT) (Figure 2) and magnetic resonance imaging (MRI) studies (Figure 3) demonstrated a large multilocular cystic lesion (6.6 × 4.2 × 5.8 cm) occupying most of the right hemithorax, mainly its posterior aspect. The mediastinum was displaced to the left, but the blood vessels and bronchi in the right hilum were not compressed. No neuroforaminal involvement was seen as well. A right thoracotomy was performed revealing a few large cysts placed in the intrapleural space as well as a few small extrapleural cysts (Figure 4). The masses were completely resected with a minimal extraction of the lung tissue adherent to the tumors. Histopathological examination revealed a multicystic tumor lined by a single layer of flat endothelial cells (Figure 5(a)), filled with proteinaceous fluid containing lymphocytes (Figure 5(b)). The cyst wall was composed of loose and dense collagenous tissue punctuated by small lymphoid aggregates. The endothelial lining cells were found diffusely positive for D2-40, lymphatic endothelial marker (Figure 6(a)). The outer surface of the cyst was partially covered by mesothelium, as highlighted by immunostaining with calretinin (mesothelial marker, Figure 6(b)). The overall histological findings fit well with a diagnosis of pleural-based multicystic lymphangioma.

The postoperative course was uneventful without any complications. Today, one year after the surgery, the child feels well and his chest radiography is normal, without evidence of recurrence of the disease.

## 3. Discussion

We reported here a case of pleural-based intrathoracic multicystic lymphangioma in a 4-month-old infant which was completely resected by surgery.

Lymphangiomas are focal proliferation of well-differentiated lymphatic tissues which are presented as a cystic, fluctuant, painless, and asymptomatic benign masses. Lymphangiomas comprise less than 5% of all vascular tumors [3]. They can affect any site of the body. However, the most common sites (75% of the cases) are the head and neck region, where the tumor is generally named “cystic hygroma.” The second most common region is the axilla including around 20% of the cases. The other sites are the thorax (1%), the retroperitoneum, the pelvis, and the groin [3, 5]. Lymphangioma of the thorax is most commonly found in the mediastinum but can also appear in the form of chest wall lesions [5], solitary pulmonary presentations [3, 6], and pleural based [2, 4]. Pleural-based lymphangioma, to the best of our knowledge, have been reported only twice in the literature. The first case described a 16-year-old teenager whose clinical presentation mimicked left pneumonia. A resection of the mass was performed, and the pathologic examination confirmed the diagnosis of pleural-based pulmonary cystic lymphangioma of the anterior mediastinum. The second case report was a 38-year-old outpatient woman with a chronic arm and shoulder pain. Positron emission tomography (PET) scan revealed that the lesion was highly fluorodeoxyglucose-avid. Biopsy exposed benign tissue consistent with solitary pleural-based pulmonary lymphangioma.

Surgical resection is the recommended treatment in lymphangioma: firstly, in order to relieve pressure from the adjacent organs; secondly, in order to confirm the diagnosis due to the difficulty in differentiating lymphangioma from other lesions such as CPAM (congenital pulmonary airway malformation), bronchogenic cysts, and the cystic form of neuroblastoma [2]. Recurrence after resection may occur as result of autonomous growth and the reaccumulation of lymphatic fluid [2]. It often appears within 3 months following the procedure though late recurrence was described even after 7 years [5]. In these cases, other types of adjuvant treatment may be proposed, such as radiotherapy, laser therapy, or injection of sclerosing agents like bleomycin, sterile ethanol, hypertonic (50%) glucose solution, fibrin glue, and a streptococcal derivative called OKT-432 [7, 8]. Since these treatments are not always successful, they are mainly reserved for inoperable cases [2, 7–9]. Systemic chemotherapy and interferon-*α* have also been experimented with limited success [5]. In our case, the tumor was large, symptomatic, and resectable. Therefore, operation was performed. Complementary therapies were unnecessary since the tumor was totally removed and no recurrences were detected in the follow-up period.

In summary, we have presented the first case of pleural-based intrathoracic multicystic lymphangioma in an infant presenting as pneumonia with complete cure after surgical resection.
